# Health demographic surveillance in Africa and South Asia – in depth network

**DOI:** 10.1186/1753-6561-7-S5-O10

**Published:** 2013-08-30

**Authors:** S Hirve

**Affiliations:** 1 Global Influenza Programme, WHO, Geneva; 2 INDEPTH Network, Board of Trustees, Switzerland

## 

The International Network for the Demographic Evaluation of Populations and Their Health in Developing Countries (INDEPTH) is a global network of members who conduct longitudinal health and demographic evaluation of populations in low- and middle-income countries. While a traditional cohort study is focused with a specific research question, the INDEPTH network aims to have a diverse portfolio by monitoring health and demographics of a fairly well defined population. The network aims to support and strengthen the ability of INDEPTH sites to conduct longitudinal health and demographic studies in defined populations, to facilitate the translation of findings to maximise impact on policy and practices, to facilitate and support research capability strengthening and to develop best practice methods for sharing and analysis of demographic and health data from multiple Health and Demographic Surveillance Systems (HDSS) sites.

Ongoing INDEPTH cross-site research activities pertain to causes of death in developing countries, NCD risk factor surveillance in Asia, adult health & ageing and indoor air pollution. The NCD risk factor surveillance involves nine HDSS sites in Asia and uses the WHO STEPS approach. The challenges faced include sustaining HDSS by reducing costs, data management, data harmonization and data sharing, ethical dilemmas of consent, population fatigue with no immediate benefit, attrition of cohorts and migration. There are also challenges of ensuring national representativeness of the data.

